# Subacute Toxicity Evaluation of Methanolic Extract of *Syzygium polyanthum* in Rats

**DOI:** 10.21315/tlsr2021.32.2.5

**Published:** 2021-06-29

**Authors:** Nor Hazliana Harun, Nor Atiqah Rasdi, Rabeta Mohd Salleh, Sabreena Safuan, Wan Amir Nizam Wan Ahmad, Wan Ezumi Mohd Fuad

**Affiliations:** 1 School of Health Sciences, Universiti Sains Malaysia, Health Campus, 16150 Kubang Kerian, Kota Bharu, Kelantan, Malaysia; 2 Food Technology Division, School of Industrial Technology, Universiti Sains Malaysia, 11800 USM Pulau Pinang, Malaysia

**Keywords:** *Syzygium polyanthum*, Sinusoidal Dilatation, Subacute Toxicity, Methanolic Extract, *Syzygium polyanthum*, Dilatasi Sinusoid, Ketoksikan Subakut, Ekstrak Metanol

## Abstract

*Syzygium polyanthum* (Wight) Walp. var. *polyanthum* (serai kayu) leaves is a popular herb and widely used in traditional medicine. Despite the ethnomedicinal benefits, very limited studies have researched on the toxicity of this plant. The aim of the present study was to investigate the potential effects of methanolic extract of *Syzygium polyanthum* (MESP) leaves via 28-day repeated oral dosing in Sprague Dawley rats. MESP leaves was administered at doses of 0 (control), 400, 1000 or 2000 mg/kg to an equal number of male and female rats (*n* = 10/group). Results obtained indicated that MESP did not affect the general conditions (body weight, feed intake and oestrous cycle) and apparent behavioural changes of the rats. Biochemical parameters revealed a slight significant variation in the aspartate aminotransferase (AST) level between the male rats treated with the lowest and highest doses of MESP, but these findings were both statistically insignificant when compared to the control group. The liver of the males (dose 1000 and 2000 mg/kg/day) also exhibited histoarchitectural defects on the hepatocytes and cytoplasm when compared to those of the control group. In contrast, female rats did not encounter any significant findings in all parameters tested. In conclusion, this study suggests that the MESP leaves might exhibit sex-based variation effects and thus, the use of this extract particularly at higher doses should be thoroughly considered.

## Abstract (fr)

Daun *Syzygium polyanthum* (Wight) Walp. var. *polyanthum* (serai kayu) ialah herba popular dan banyak digunakan dalam perubatan tradisional. Walaupun terdapat manfaat etnoperubatan, kajian ke atas ketoksikan tumbuhan ini sangat terhad. Tujuan kajian ini adalah untuk mengkaji kemungkinan kesan ekstrak metanol daun *Syzygium polyanthum* (MESP) melalui dos oral berulang 28 hari ke atas tikus Sprague Dawley. MESP diberikan pada dos 0 (kawalan), 400, 1000 atau 2000 mg/kg kepada tikus jantan dan betina yang sama bilangan (*n* = 10/kumpulan). Hasil yang diperolehi menunjukkan bahawa MESP tidak mempengaruhi keadaan fizikal (berat badan, pengambilan makanan dan kitaran estrus) dan perubahan jelas ke atas tingkah laku tikus. Parameter biokimia menunjukkan sedikit perbezaan yang signifikan dalam aras aspartate aminotransferase (AST) di antara tikus jantan yang dirawat dengan dos MESP terendah dengan dos tertinggi, tetapi keputusan ini tidak signifikan secara statistik bila dibandingkan dengan kumpulan kawalan. Hepar tikus jantan (dos 1000 dan 2000 mg/kg/hari) juga menunjukkan kecacatan histoarkitek pada hepatosit dan sitoplasma bila dibandingkan dengan kumpulan kawalan. Sebaliknya, tikus betina tidak menunjukkan keputusan yang signifikan dalam semua parameter yang diuji. Kesimpulannya, kajian ini mencadangkan bahawa MESP mungkin menunjukkan perbezaan kesan berdasarkan jantina dan oleh itu, penggunaan ekstrak ini terutama pada dos tinggi harus dipertimbangkan sewajarnya.

**Highlights**

- Oral treatment of methanolic extract of *Syzygium polyanthum* leaves (MESP) particularly the higher dose (1000 and 2000 mg/kg/day) groups demonstrated increment of aspartate aminotransferase (AST) level and histoarchitectural defects on the liver of male animals.
- All female rats treated with MESP did not possess any definite toxicological effects.
- MESP leaves might exhibit sex-based variation effects.

## INTRODUCTION

Natural products have been widely utilised as the main materials and played a crucial role as the lead compound in the development of a new drug. Recently, the exploitation of medicinal herbs as alternative medicines in curing various types of diseases and infections has exponentially increased. This is due to the conservative claim that these products are safe and free from side effects as been embraced in the ancient traditional Chinese, Greek-Unani and Ayurveda medicines (Patwardhan *et al*. 2005).

*Syzygium polyanthum* (Wight) walp. var. *polyanthum* belongs to *Myrtaceae Juss* family, which is a flowering plant that is widely distributed throughout Asia including Myanmar, Indo-China, Thailand, Malaysia and Indonesia (Java, Sumatera and Kalimantan). This plant has various vernacular names such as *ubar serai* and *meselengan* in Sumatera; *samak, kelat samak* and *serah* in Malaysia; *manting* in Java (Agoes 2010) whereas it is natively known as *serai kayu* or *salam* by the local populace in Kelantan, Malaysia. Besides its vernacular name, this plant is also recognised globally as Indian or Indonesian bay leaves. In Malaysia, *S. polyanthum* leaves have been traditionally used as remedy for various illnesses such as diarrhoea, gastritis, hypertensive, hypercholesterolemia, cataracts, diabetes mellitus and skin diseases (Sumono & Wulan 2008). The young shoots are exotic spice that can be consumed as fresh salad (*ulam*) and the mature leaves are used as flavour enhancer in Indonesian and Malay cuisines such as *nasi kerabu* and Kelantanese *laksam* (Ismail *et al*. 2013).

Despite the popular uses of *S. polyanthum* leaves in treating illnesses, there is still lack of scientific data available on the safety profiles of this herb with regards to its toxicological facts. Recently, Sumiwi *et al*. (2019) had completed the subchronic toxicity study of ethanol extract of this plant in rats for 90 days of treatment period. This study revealed that the plant extract did not show toxicity on most parameters, but fatty liver and necrosis were observed in female rats. Previously, an acute toxicity assessment of aqueous extract of *S. polyanthum* mixed with *Andrographis paniculata* leaf was conducted by Widharna *et al*. (2015). This experiment indicated that a single dose of 2000 mg/kg body weight of this plant extract did not exhibit acute signs of toxicity in all rats. Apart from this, the acute toxicity study of methanol extract of *S. polyanthum* leaves had also been performed by our research group. This preliminary experiment proved that *S. polyanthum* extract at a dose of 2000 mg/kg did not show acute deleterious effect in rats (data not published). However, only these few studies conducted are still not adequate to justify the broader safety information of this herb as well as in gaining trust for its quality prior to be developed as a new herbal medicinal product.

Recognising the popular nature of *S. polyanthum* as an edible species and couple with the fact that there still a dearth of information on its safety information, it is therefore necessary that the subacute oral toxicity study was carried out to investigate the potential toxicological effects of this herb on 28 days repeated dosing in both male and female rats. The present toxicological study encompasses a wide range of assessment and end-points which collectively can demonstrate potential impairment *in vivo*. This experiment is relevant to step up emphasising the safety profiles and help to provide additional supporting evidence on the potential effects of this plant on an animal model. Information gathered could be utilised as an initial scientific platform and basis for the initiation of other related works investigating the potential toxicological effects of other herbal preparations. Apart from expanding new knowledge regarding this herb, the present findings can also indirectly enlighten consumers on the importance of utilising safe products while ultimately will help to protect the well-being of the public.

## MATERIALS AND METHODS

### Collection of Plant Materials

The old leaves of *S. polyanthum* were collected from the district of Ketereh, Kelantan, Malaysia. The plant was identified by the ethnobotanist, Dr. Richard Chung from the Forest Research Institute Malaysia (FRIM). The herbal specimen (dried leaves) was deposited in the herbarium of FRIM with sample number: PID-171011-10.

### Plant Extraction

The methanol extract of *S. polyanthum* (MESP) leaves was prepared in the Biomedicine Laboratory, School of Health Sciences, Universiti Sains Malaysia (USM) Health Campus, Kubang Kerian, Kelantan. Prior to the extraction process, the plant leaves were weighed, washed with distilled water and dried in an incubator at a pre-set temperature of 50°C for four consecutive days.

The dried leaves were subsequently ground into powder form using a laboratory grinder and filtered using a stainless-steel sieve. The total weight of powder was then measured using a digital weighing balance. Later, about 150 g of *S. polyanthum* leaves powder was immersed in 350 mL of n-hexane and sonicated for about 30 min. The solution was filtered using Whatman Paper No. 1 and the residue was put under fume hood until n-hexane was completely evaporated. The similar above-mentioned steps were then repeated using ethyl acetate. After the ethyl acetate has completely evaporated, the residue was then immersed in methanol at three different cycles: 400 mL, 200 mL and 50 mL. The solution from each methanol cycle was also filtered using the Whatman Paper No. 1. The remaining plant residue was discarded while the filtered supernatant was evaporated using rotary evaporator at 42°C–45°C until the solvent totally be removed. The weight of the crude dried extract was measured using digital weighing balance. Prior to use, the extract was dissolved with 1% of carboxymethylcellulose (CMC) sodium salt in distilled water.

### Dosage Preparation

The selection of MESP doses for this present study was based on the previous preliminary toxicological study performed by our research group (data not published) to find the suitable dose range involving a low, medium and high dose for *in vivo* experiment [Organisation for Economic Co-Operation and Development (OECD) 2008]. The preliminary study manipulated the pharmacological dose (20 mg/kg–100 mg/kg) of MESP leaves obtained from our team, Ismail *et al*. (2013). This was set at three different dosages of 400, 1000 and 2000 mg/kg/day whereas distilled water (DW) was used as vehicle. Two days prior to administration of extract to the test animals, the MESP powder was weighed based on the average body weight of rats and reconstituted with approximately 0.8 mL–1.0 mL of distilled water to become a fixed, total volume of 1.5 mL. The MESP solution was then homogenised with a homogeniser at 24,000/min for 3 min to ensure a uniform distribution of extract particles. The prepared doses were stored in the refrigerator at −20°C until use.

### Animal Husbandry and Maintenance

The approval for this study was obtained from the USM Institutional Animal Care and Use Committee (IACUC) with the reference number: USM/Animal Ethics Approval/2015/(701)). Thirty male and 30 female Sprague Dawley rats (2–3 months old), weighing 200 g–220 g and 180 g–200 g respectively were used in this experiment. The selection of this range of age and weight was due to the suitable physiological and reproductive characteristics of fertile, young adult of this rat strain (Sengupta 2013). These animals were acquired from the Animal Research and Service Centre (ARASC), USM Health Campus, Kubang Kerian, Kelantan. All animals were individually placed in cages and kept in the ARASC animal room. They were maintained under standard laboratory conditions including the temperature of 22 ± 3°C, the humidity within 50%–60% and the 12 h:12 h light-dark cycle (lights on from 0700 to 1900 hrs). The animals were fed with adequate amount of standard commercial rat pellet (Gold Coin Feedmills, Malaysia) diet of 19 g per day except during food fasting and unlimited supply of water *ad libitum*. Prior to experiment, animals were acclimatised for a week.

### Experimental Protocol and Dose Selection

Prior to dosing, all animals were examined for any physical and behavioural abnormalities whereas food consumption and body weights were measured daily. Moreover, female rats were observed for their oestrous cyclicity by performing vaginal smears at around the same time every day. The procedure of obtaining vaginal smear in this study was based on Wan Ezumi *et. al*. (2017). Those possessed with abnormal patterns or irregular length of cycle or health deficit were excluded from the study.

Adapting the OECD Guideline No. 407 (OECD 2008), 10 SD rats that comprised of five males and five females were assigned to each dose level; 0 (distilled water - control), MESP 400, 1000 and 2000 mg/kg body weight in a total volume of 1.5 mL each. The treatment of rats began on a day after they enter the age of nine weeks. All treatments were orally administered by gavaging once per day for a period of 28 days. For female rats, the dose administration began after they entered dioestrus stage of the oestrous cycle.

All experimental rats were fasted overnight for 16 h from the evening of the day before the scheduled sacrifice. At autopsy, as soon as the rat achieved a state of anaesthesia, blood sample was collected from the abdominal aorta for further analysis. The male rats were sacrificed on day 29 of experimental period. In contrast, the laparotomy of female rats could be delayed up to several days until the rats reached the dioestrus stage (MESP administration were sustained) to standardise the timing of decease of all females. This is important to normalise the diurnal hormonal variation in the females particularly when effects on the reproductive organs are concerned (OECD 2008; Wan Ezumi *et al*. 2017).

### Haematology

During blood collection of a rat, approximately 2 mL of blood was withdrawn from the abdominal aorta using a syringe. The blood was kept in heparinised EDTA tubes for the measurement of haematological parameters. These included analysis on total red blood cell count (RBC), haemoglobin, red blood cell indices (mean corpuscular volume (MCV), mean corpuscular haemoglobin (MCH), mean corpuscular haemoglobin concentration (MCHC), red blood cell distribution width (RDW), total white blood cell count (WBC), WBC differential count (polymorph nuclear cells, lymphocytes, monocytes, eosinophils and basophils), platelet count (PC), serum T4 and phosphorus (BP Clinical Laboratory Service Guide 2015).

### Serum Biochemistry

Approximately 3 mL of blood withdrawn from each animal was extracted by centrifugation at 1800 x g for 15 min to obtain serum. The serum collected were analysed for sodium, potassium, chloride, calcium, total protein, albumin, total bilirubin, bilirubin, creatinine, blood urea nitrogen (BUN), uric acid, cholesterol (total, high-density lipoprotein (HDL) and low-density lipoprotein (LDL), triglycerides, alkaline phosphatase (ALP), gamma-glutamyltransferase (GGT), serum glutamic oxaloacetic transaminase (SGOT) and serum glutamic-pyruvic transaminase (SGPT) (BP Clinical Laboratory Service Guide 2015).

### Gross Assessment and Histopathology

Following blood collection, all animals were immediately sacrificed by aortic exsanguinations. They underwent detailed gross necropsy which included careful examination of external surfaces of the body, all orifices, cranium, thorax and abdominal cavities with their contents. Subsequently, visceral organs and tissues were removed from the body, cleared of any adhering tissues, blotted dry from normal saline and weighed (to get both absolute and relative weights). Target organs including the liver, kidney, spleen, adrenals and reproductive organs (testes/ovaries) were then fixed in 10% neutral buffered formalin or Bouin’s solution (testes) prior to histological procedures.

### Statistical Analysis

All data were analysed using GraphPad-Prism version 6.0.1 software (GraphPad, San Diego, CA). Data were submitted for test of normality using descriptive statistics. Two-way ANOVA was used followed by Bonferroni post-hoc test to analyse body weight and food consumption. Additionally, one-way ANOVA followed by Bonferroni post-hoc test was used to analyse absolute and relative organ weights data, haematological and biochemistry parameters. Further, non-parametric of Kruskal-Wallis test followed by Mann-Whitney U test (when appropriate) was applied to analyse the length of oestrous cycle which was between 4–6 days for each female. The data were expressed as mean ± standard error of mean (S.E.M) for parametric data while median and interquartile range (IQR) for non-parametric data. The level of probability, *p* < 0.05 was considered as significant.

## RESULTS

### Morbidity and Mortality of Rats

There were no treatment related morbidity and mortality associated with the adverse effect in rats at any dose level tested throughout the administration of MESP leaves for 28 days.

### General Observations and Behavioural Changes

Most of treated rats were observed to be in normal condition during treatment periods. There were no abnormalities with regards to the skin, fur, eye colour, nasal discharge and faeces, locomotor progression and respiration. A few rats that received the highest dose of MESP leaves (2000 mg/kg/day) however displayed a weird circling motion in the cages soon after gavaging were made (during first few days of treatment only) due to bitter taste of the extract.

### Body Weight

During 28 days treatment period, all rats gained their body weights normally. Their increased pattern of weight were comparable to each other according to sexes.

### Food Consumption

The food consumption of all animals was measured and compared to each other according to sexes throughout the experimental period. All male and female rats both the control and treated animals showed no significant differences in the food intake within 28 days treatment period.

### Oestrous Cycle

The administration of MESP leaves did not adversely alter the oestrous cyclicity of female rats in this study. The average length of their oestrous cycle were between 4 to 6 days and revealed no significant differences (*p* > 0.05) between the control and treatment groups.

### Clinical Haematology

The administration of herbal treatment for 28 days in this study exhibited non-significant (*p* > 0.05) effect in the values of all haematological parameters for both sexes of animals.

### Clinical Biochemistry

Subacute oral administration of MESP leaves to rats revealed no statistically significant differences in the values of all biochemical parameters tested except for AST. The AST levels were found to be significantly (*p* < 0.05) elevated in the male rats of Group 4 (194.60 ± 33.91) when compared with the Group 2 (107.40 ± 7.88) (Table 1). However, these findings were found to be not significant (*p* > 0.05) when compared to the control group.

### Gross Examination

The inspection performed at autopsy revealed that there was no treatment related gross findings in all rats of any dosage groups. All external surfaces of the body, all orifices, cranium, thorax and abdominal cavities with their contents demonstrated no changes for both control and treated animals. All visceral organs including the liver, kidney, spleen, adrenals and reproductive organs (testes, ovaries) were uniformly healthy and appeared to be in normal shapes, sizes, positions and colours. Furthermore, no significant morphological or haemorrhagic changes were observed on these organs due to the administration of MESP leaves.

### Absolute and Relative Organ Weight

There were no significant differences in the absolute and relative organ weights of both male and female animals when compared to their respective control groups. Table 2 presents the relative organs weights of the animals.

### Histopathology of Organs

Even though none of the visceral organs showed statistical differences in their absolute and relative weights, but histopathological findings of the liver of male rats particularly, showed some alterations. Meanwhile, there were no apparent abnormalities were noted on the histology of the kidney, spleen, adrenals and testes, they were therefore not discussed further in this article. Moreover, the histological findings of the females’ internal organs (liver, kidney, spleen, adrenal glands and ovaries) also did not exhibit any apparent abnormalities caused by the subacute treatment with MESP leaves.

**Figure 1:**
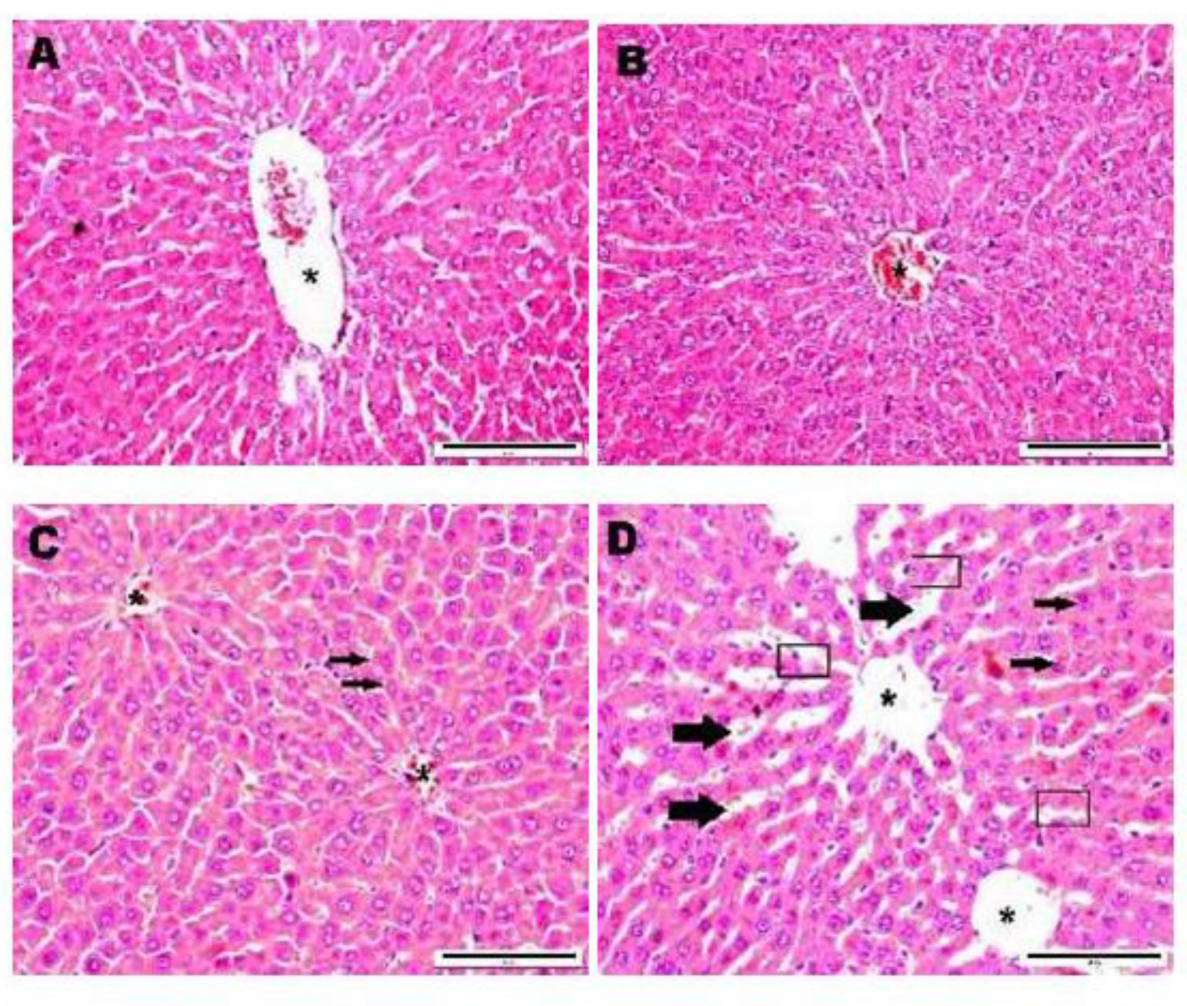
Photomicrograph showing the histological sections of liver tissue of male rats (20x magnification). (A) Group 1 (control-DW), (B) Group 2 (MESP 400 mg/kg), (C) Group 3 (MESP 1000 mg/kg), (D) Group 4 (MESP 2000 mg/kg) (*: Central vein; □: Denudation of epithelial lining; → : Hepatocyte degeneration; ➡ : Sinusoidal dilatation)

Microscopically in male rats, histological features of the liver of control (0 mg/kg; Group 1) and the lowest dose of MESP (400 mg/kg; Group 2) showed normal structures (Figs. 1A and 1B). However, some defects were noted on the histoarchitecture of liver hepatocytes of rats treated with the middle range dose (1000 mg/kg; Group 3) and the highest dose (2000 mg/kg; Group 4) (Figs. 1C and 1D). The defects observed include increased sinusoidal space, denudation of epithelial lining and cellular hepatocytes degeneration. Mild necrosis was also found in the centrilobular regions of the liver of male rats (2000 mg/kg; Group 4). In contrast, the dosing with MESP did not produce any deleterious effect on the histological evaluation of liver for all female rats.

## DISCUSSION

*Syzygium polyanthum* is one of the promising herbs that possesses wide pharmacological properties. However, there are still limited scientific toxicological data available for this plant. In line with this scenario, the present subacute (28 days) toxicity study was conducted by adopting procedures of the OECD Guideline No. 407 (OECD, 2008) to determine the potential short term toxicological effects of MESP leaves in order to support its safety profiles prior to be developed as candidate of modern medicinal products. The usual duration of treatment for subacute toxicity studies is 14 days–28 days (2 weeks–4 weeks) with the general aim of finding a dose range to be used in subsequent subchronic and chronic toxicity studies (Denny & Stewart 2017).

The current study utilised methanol extract of SP leaves because it gave a greater effect in extracting polar compounds when compared to aqueous solvent, besides producing more extraction yield (Do *et al*. 2014). Following 28 days treatment period, findings obtained have shown that there were no MESP leaves-related morbidity, mortality and any apparent behavioural changes to all animals at any dose levels tested. Even though a few animals receiving the highest dose of MESP leaves (2000 mg/kg/day) displayed weird motion for few seconds soon after gavaging, but this effect was due to bitter taste of the extract and temporarily observed during the first few days of treatment only.

The healthy patterns of food intake with the normal increment of body weight of all groups of animals were also seen throughout the study. Generally, the reduction in body weight gain is a simple yet a sensitive indicator in toxicity studies after being exposed to any potentially toxic substances (Nirogi *et al*. 2014). Extensively, body weight parameter has been used in assessing the cause of the disease and the response towards therapy of drugs (Barbosa-da-Silva *et al*. 2012).

During laparotomy, the macroscopical examinations of the organs of rats treated with MESP leaves did not exhibit any abnormal appearance with regards to their size, colour and surface texture as compared to those of the control group. Moreover, no signs of organ hypertrophy were observed in all animals, by which the hypertrophy of organ is considered as one of the important toxicity endpoints following exposure to any xenobiotics (Hall *et al*. 2012). Correspondingly, weighing of organs has conventionally been used in providing a useful indication to identify a target organ prior to performing histopathology. As widely established, internal organ weight plays an important role in toxicology since alteration in the weight could reflect organ toxicity (Sellers *et al*. 2007). As found in this study, both absolute and relative organ weights were statistically comparable among all groups of animals. The information obtained from both weights are crucial for prediction of toxicity but however, the relative weight which is the normalisation of organ weights to body weight is more optimum than absolute weight (Nirogi *et al*. 2014).

Parameters of clinical pathology that include clinical biochemistry and haematology are the standard criteria monitored during toxicity studies to provide valuable information regarding the overall health status, general metabolic, mechanism of toxicity, and target organs (Everds 2015). Exposure of male rats to MESP leaves in this study has shown that there were slight changes in the biochemical parameter particularly the AST concentration between the two treated groups. However, these findings were comparable with the vehicle control group. Additionally, the AST levels of the males in both Groups 2 and 4 of this current experiment were still within the normal range for male Sprague-Dawley rats (Park *et al*. 2016). In toxicity studies, it is necessary to report the findings even though not significantly different from the control animals (Hamada 2018). The elevations of enzymes such as AST or serum glutamate oxaloacetic transaminase (SGOT) level in the blood indicates cellular leakages and loss of functional integrity then disruption of cell membrane in liver tissue (Hilaly *et al*. 2004; McGill 2016). Primarily, ALT can be found in the liver while AST is distributed in a wide variety of tissues other than the liver (hepatocytes) including the heart, skeletal muscle, kidney, and brain (Thapa & Walia 2007; Aulbach & Amuzie 2017). Based only on this AST value, it would be suggested that the subacute administration of MESP leaves did not cause serious impairment to the enzymes system of rats. However, these would be further verified by the histological examination on the related organs to substantiate the findings.

Approaching the end of this study, the macroscopical evaluation of MESP leaves was conducted on all organs but only certain target organs were assessed histopathologically. Histopathology is an evergreen yet the gold standard for identifying a treatment-related effect on any organ. The advantages of this technique are it can provide a more comprehensive view of disease and its effect on tissues, since the preparation process preserves the underlying tissue architecture (Gurcan *et al*. 2009). Among other organs, both the liver and kidney were chosen because they have a high affinity for toxic substances and store more toxicants than any tissues in the body (Mohamed *et al*. 2011; Yuet *et al*. 2013). According to David and Hamilton (2010), hepatic damage is one of the vital consequences resulted from xenobiotics and drugs administration. Hence, it is very crucial to confirm whether *S. polyanthum* leaves may cause possible damage to the visceral organs.

Our present findings demonstrated that no treatment-related toxicological significances were observed in the histological sections of the kidney, spleen, adrenals, and reproductive organs (testes/ovaries) for all animals examined. Nonetheless, the liver sections of males administered with the middle (1000 mg/kg; Group 3) and the highest doses (2000 mg/kg; Group 4) of MESP leaves displayed some abnormal morphology when compared to those of the control group. In contrast, the liver sections of male rats in the lowest dose group (MESP 400 mg/kg; Group 2) were histologically normal, comparable to the control.

In these particular dose groups (1000 and 2000 mg/kg), the liver samples displayed enlargement of the nucleus. This similar incidence is often seen in rats treated with xenobiotics. The sinusoidal dilatation was also noted and more prominent in the centrilobular region (Zone 3) when compared with the liver section of control group. The damage occurred in Zone 3 could result in greater alteration of AST levels, as noticed in the male rats treated with MESP 2000 mg/kg. In addition, the liver tissue of animals receiving the same dose also showed histological changes, i.e., the presence of denuded epithelial lining. Pericentral zone or centrilobular region is the first target area to experience hypoxic stress and xenobiotic metabolism as it lacks oxygen supply (Will *et al*. 2016). Hepatic sinusoidal dilatation (HSD) or sinusoidal dilatation and congestion (SDC) is a condition resulted from venous outflow obstruction, infectious condition, or infiltration of sinusoids by various types of benign or malignant cells (Saadoun *et al*. 2004). In contrast, the histological assessment of the female livers did not reveal any deleterious effect after being treated with MESP leaves.

## CONCLUSIONS

Taking all data together, this study revealed that oral treatment of MESP leaves particularly the higher dose groups demonstrated vital pharmacological response i.e histoarchitectural defects on the liver of male SD rats. Nevertheless, all female rats did not possess any definite toxicological effects from MESP treatment. Therefore, this study suggests that the MESP leaves might exhibit sex-based variation effects and thus, the use of this extract particularly at higher doses should be thoroughly considered.

## Figures and Tables

**Table 1:** Effect of MESP leaves onterminal clinical biochemistry of SD rats for subacute (28 days) toxicity study.

Parameters	Group 1 (control)	Group 2 (400 mg/kg)	Group 3 (1000 mg/kg)	Group 4 (2000 mg/kg)	*p*-value
Males					
Sodium (mmol/L)	144.00 ± 0.77	144.00 ± 0.55	145.60 ± 0.81	144.20 ± 0.37	n/s
Potassium (mmol/L)	6.19 ± 0.33	6.01 ± 0.12	6.86 ± 0.69	6.86 ± 0.49	n/s
Chloride (mmol/L)	101.80 ± 0.37	100.80 ± 0.73	101.40 ± 1.21	101.20 ± 0.66	n/s
Creatinine (umo/L)	41.72 ± 1.65	45.76 ± 1.08	46.98 ± 1.64	44.02 ± 1.47	n/s
BUN (mg/dL)	22.00 ± 1.14	21.40 ± 1.12	22.80 ± 1.07	19.60 ± 1.40	n/s
Total cholesterol (mmol/L)	1.76 ± 0.04	1.68 ± 0.04	1.82 ± 0.09	1.62 ± 0.06	n/s
HDL (mmol/L)	0.90 ± 0.12	0.94 ± 0.10	0.98 ± 0.09	0.96 ± 0.05	n/s
LDL (mmol/L)	0.66 ± 0.12	0.54 ± 0.10	0.54 ± 0.09	0.46 ± 0.05	n/s
Triglyceride (mmol/L)	0.40 ± 0.06	0.44 ± 0.02	0.62 ± 0.12	0.40 ± 0.03	n/s
Total protein (g/L)	54.20 ± 1.83	55.40 ± 0.75	56.60 ± 1.21	54.00 ± 0.71	n/s
Albumin (g/L)	26.80 ± 0.37	26.40 ± 0.24	26.40 ± 0.51	25.40 ± 0.68	n/s
Total bilirubin (umo/l)	1.70 ± 0.00	1.70 ± 0.00	1.70 ± 0.00	1.70 ± 0.00	n/s
AST (U/L)	155.80 ± 24.07	107.40 ± 7.88^a^	121.20 ± 3.89	194.60 ± 33.91^a^	*p* < 0.05
ALT (U/L)	63.40 ± 6.52	59.00 ±1.26	63.80 ± 5.00	67.60 ± 12.03	n/s
GGT (U/L)	4.20 ± 0.20	4.00 ± 0.00	4.00 ± 0.00	4.00 ± 0.00	n/s
ALP (U/L)	204.00 ± 19.83	226.40 ± 10.64	251.60 ± 41.72	154.60 ± 18.62	n/s
Calcium (mmol/L)	2.24 ± 0.04	2.28 ± 0.02	2.56 ± 0.15	2.42 ± 0.06	n/s
Phosphorus (nmol/L)	3.30 ± 0.10	3.35 ± 0.13	3.60 ± 0.28	3.34 ± 0.15	n/s
Females					
Sodium (mmol/L)	139.80 ± 1.16	143.60 ± 0.51	141.60 ± 0.75	140.60 ± 1.29	n/s
Potassium (mmol/L)	6.94 ± 0.60	6.68 ± 0.90	6.22 ± 0.70	6.12 ± 0.76	n/s
Chloride (mmol/L)	96.60 ± 1.86	99.40 ± 1.33	99.60 ± 0.93	100.00 ± 1.05	n/s
Creatinine (umo/L)	48.58 ± 1.99	49.82 ± 1.39	47.18 ± 2.27	49.44 ± 1.93	n/s
BUN (mg/dL)	23.80 ± 3.14	23.40 ± 1.81	22.00 ± 2.37	21.00 ± 1.70	n/s
Total cholesterol (mmol/L)	2.10 ± 0.10	2.02 ± 0.10	2.10 ± 0.19	1.88 ± 0.12	n/s
HDL (mmol/L)	1.40 ± 0.08	1.34 ± 0.10	1.38 ± 0.10	1.24 ± 0.09	n/s
LDL (mmol/L)	0.46 ± 0.07	0.46 ± 0.05	0.46 ± 0.09	0.42 ± 0.05	n/s
Triglyceride (mmol/L)	0.46 ± 0.04	0.50 ± 0.08	0.50 ± 0.06	0.42 ± 0.06	n/s
Total protein (g/L)	61.40 ± 0.81	60.60 ± 0.81	61.80 ± 2.24	62.00 ± 0.63	n/s
Albumin (g/L)	29.00 ± 0.05	28.00 ± 0.55	27.80 ± 0.73	27.00 ± 0.71	n/s
Total bilirubin (umo/l)	1.70 ± 0.00	1.70 ± 0.00	2.04 ± 0.34	2.04 ± 0.34	n/s
AST (U/L)	119.80 ± 5.25	136.00 ± 4.88	121.80 ± 5.12	171.00 ± 31.85	n/s
ALT (U/L)	50.20 ± 3.38	59.40 ± 6.14	52.40 ± 4.62	64.40 ± 3.64	n/s
GGT (U/L)	4.00 ± 0.00	4.00 ± 0.00	4.40 ± 0.40	4.00 ± 0.00	n/s
ALP (U/L)	162.60 ± 22.48	194.00 ± 44.26	161.60 ± 20.92	129.20 ± 15.05	n/s
Calcium (mmol/L)	2.34 ± 0.04	2.42 ± 0.08	2.40 ± 0.03	2.46 ± 0.04	n/s
Phosphorus (nmol/L)	3.90 ± 0.90	2.86 ± 0.27	2.66 ± 0.24	2.64 ± 0.16	n/s

*Note*: Data are expressed as mean ± SEM. n/s = Statistically not significant.

a *p <* 0.05 when compared between Group 2 (400 mg/kg) and Group 4 (2000 mg/kg) but n/s (*p >* 0.05) with the control group.

**Table 2:** Effect of MESP leaves on relative organ weights of SD rats for subacute (28 days) toxicity study.

Parameters (g/100g)	Group 1 (control)	Group 2 (400 mg/kg)	Group 3 (1000 mg/kg)	Group 4 (2000 mg/kg)	*p*-value
Males					
Brain	0.71 ± 0.03	0.69 ± 0.02	0.68 ± 0.01	0.73 ± 0.01	n/s
Heart	0.34 ± 0.01	0.34 ± 0.01	0.36 ± 0.02	0.34 ± 0.01	n/s
Lung (R)	0.29 ± 0.01	0.31 ± 0.02	0.30 ± 0.01	0.32 ± 0.00	n/s
Lung (L)	0.15 ± 0.00	0.17 ± 0.01	0.17 ± 0.01	0.17 ± 0.00	n/s
Liver	3.25 ± 0.07	3.29 ± 0.06	3.26 ± 0.11	3.08 ± 0.04	n/s
Spleen	0.29 ± 0.02	0.29 ± 0.01	0.28 ± 0.01	0.29 ± 0.02	n/s
Kidney (R)	0.36 ± 0.02	0.35 ± 0.01	0.37 ± 0.03	0.35 ± 0.01	n/s
Kidney (L)	0.35 ± 0.02	0.31 ± 0.03	0.35 ± 0.02	0.35 ± 0.02	n/s
Adrenal gland (R)	0.01 ± 0.00	0.02 ± 0.01	0.01 ± 0.00	0.01 ± 0.00	n/s
Adrenal gland (L)	0.01 ± 0.00	0.01 ± 0.00	0.01 ± 0.00	0.01 ± 0.00	n/s
Stomach	1.20 ± 0.14	1.28 ± 0.32	1.22 ± 0.27	1.06 ± 0.11	n/s
Intestines	7.32 ± 0.58	6.39 ± 0.28	6.93 ± 0.11	6.69 ± 0.17	n/s
Bladder	0.04 ± 0.00	0.03 ± 0.00	0.04 ± 0.01	0.04 ± 0.00	n/s
Seminal vesicle (R)	0.10 ± 0.01	0.12 ± 0.03	0.13 ± 0.01	0.11 ± 0.01	n/s
Seminal vesicle (L)	0.15 ± 0.02	0.13 ± 0.02	0.14 ± 0.02	0.11 ± 0.02	n/s
Prostate gland (R)	0.09 ± 0.01	0.09 ± 0.00	0.08 ± 0.00	0.09 ± 0.00	n/s
Prostate gland (L)	0.08 ± 0.00	0.09 ± 0.00	0.08 ± 0.00	0.09 ± 0.01	n/s
Testis(R)	0.52 ± 0.07	0.58 ± 0.01	0.57 ± 0.03	0.59 ± 0.01	n/s
Testis (L)	0.60 ± 0.01	0.58 ± 0.01	0.58 ± 0.04	0.59 ± 0.02	n/s
Epididymis and Vas deferens (R)	0.10 ± 0.00	0.17 ± 0.07	0.11 ± 0.01	0.11 ± 0.01	n/s
Epididymis and Vas deferens (L)	0.10 ± 0.00	0.10 ± 0.01	0.11 ± 0.00	0.11 ± 0.01	n/s
**Females**					
Brain	0.85 ± 0.02	0.86 ± 0.02	0.82 ± 0.04	0.85 ± 0.03	n/s
Heart	0.36 ± 0.01	0.35 ± 0.01	0.38 ± 0.00	0.36 ± 0.00	n/s
Lung (R)	0.26 ± 0.03	0.36 ± 0.09	0.33 ± 0.03	0.37 ± 0.01	n/s
Lung (L)	0.31 ± 0.07	0.27 ± 0.03	0.26 ± 0.06	0.19 ± 0.01	n/s
Liver	3.67 ± 0.12	3.67 ± 0.12	3.61 ± 0.12	3.39 ± 0.08	n/s
Kidney (R)	0.39 ± 0.01	0.37 ± 0.01	0.37 ± 0.01	0.38 ± 0.01	n/s
Kidney (L)	0.38 ± 0.01	0.36 ± 0.01	0.35 ± 0.01	0.36 ± 0.01	n/s
Adrenal gland (R)	0.02 ± 0.00	0.02 ± 0.00	0.02 ± 0.00	0.02 ± 0.00	n/s
Adrenal gland (L)	0.02 ± 0.00	0.02 ± 0.00	0.02 ± 0.00	0.02 ± 0.00	n/s
Spleen	0.32 ± 0.03	0.35 ± 0.01	0.31 ± 0.01	0.32 ± 0.01	n/s
Stomach	1.22 ± 0.19	1.42 ± 0.21	1.27 ± 0.25	0.85 ± 0.05	n/s
Intestine	7.88 ± 0.65	7.24 ± 0.42	7.00 ± 0.54	6.88 ± 0.33	n/s
Bladder	0.04 ± 0.00	0.04 ± 0.00	0.04 ± 0.00	0.04 ± 0.00	n/s
Uterus	0.25 ± 0.02	0.27 ± 0.02	0.24 ± 0.02	0.22 ± 0.02	n/s
Fallopian tube (R)	0.01 ± 0.00	0.01 ± 0.00	0.01 ± 0.00	0.01 ± 0.0	n/s
Fallopian tube (L)	0.01 ± 0.00	0.01 ± 0.00	0.01 ± 0.00	0.01 ± 0.00	n/s
Ovary (R)	0.02 ± 0.00	0.02 ±0.00	0.02 ± 0.00	0.02 ± 0.00	n/s
Ovary (L)	0.02 ± 0.00	0.02 ± 0.00	0.02 ± 0.00	0.02 ± 0.00	n/s

*Note*: Data are expressed as mean ± SEM. n/s = statistically not significant.
